# Integrated Evaluation of *Mentha rotundifolia* (L.) Huds Essential Oil: Physicochemical Characterization, Antibacterial Effect and In Silico ADMET Prediction

**DOI:** 10.3390/ijms27083527

**Published:** 2026-04-15

**Authors:** Meryem Benyamane, Soukaina Elorchi, Imane Brahimi, Nouhaila Belasla, Mohammed Salah, Faouzi Errachidi, Giulia Tabanelli, Vida Šimat, Fatih Ozogul, Chakib El Adlouni, Abdellah Zinedine

**Affiliations:** 1 Laboratory of Marine Biotechnologies and Environment (BIOMARE), Faculty of Sciences, Chouaib Doukkali University, P.O. Box 20, El Jadida 24000, Morocco; benyamane.meryem@ucd.ac.ma (M.B.); brahimi.imane@ucd.ac.ma (I.B.); belaslanouhaila@gmail.com (N.B.); chakib_eladlouni@yahoo.fr (C.E.A.); 2 Molecular Modeling and Spectroscopy Research Team, Department of Chemistry, Chouaïb Doukkali University, P.O. Box 20, El Jadida 24000, Morocco; elorchisoukaina2210@gmail.com (S.E.); salahmed534@gmail.com (M.S.); 3 Applied Organic Chemistry Laboratory, Faculty of Science and Technology, Sidi Mohammed Ben Abdellah University, P.O. Box 2202, Fez 30050, Morocco; errachidifaouzi@yahoo.fr; 4 Department of Agricultural and Food Sciences, University of Bologna, 40127 Bologna, Italy; giulia.tabanelli2@unibo.it; 5 Department of Marine Studies, University of Split, 21000 Split, Croatia; vida@unist.hr; 6 Department of Seafood Processing Technology, Faculty of Fisheries, Çukurova University, Balcalı, Adana 01330, Türkiye; fozogul@cu.edu.tr

**Keywords:** essential oil, *Mentha rotundifolia*, physicochemical characterization, gas chromatography–tandem mass spectrometry, antibacterial activity, molecular docking, in silico ADMET

## Abstract

This study aimed to evaluate the physicochemical characterization and antibacterial activity of the essential oil (EO) extracted from the leaves of *Mentha rotundifolia* (L.) Huds. Molecular interactions between bioactive ligand compounds, target bacterial proteins and DNA gyrase subunit B (GyrB), as well as an in silico ADMET prediction study, were also conducted. The EO was obtained by hydrodistillation of the plant leaves. The Gas Chromatography–Tandem Mass Spectrometry (GC-MS/MS) analysis revealed Rotundifolone (27.95%) and carvacrol (19.48%) as the major constituents. Other components identified included Piperitenone (6.09%), Cinerolon (4.73%), and Pulegone (4.47%). Antibacterial activity was assessed against six bacterial strains: *Enterococcus faecalis* CIP 103214, *Salmonella* Typhi CIP 5535, *Staphylococcus aureus* ATCC 9144, *Bacillus cereus* ATCC 33019, *Streptococcus agalactiae* IPM 24842, and *Providencia alcalifaciens* CIP 82.90T. The disk diffusion assay showed a strong inhibitory effect against *E. faecalis* (inhibition zone: 19.66 ± 0.3 mm), while the lowest minimum inhibitory concentration (MIC) was observed for *B. cereus* (0.58 ± 0.01 µL/mL). The time-kill kinetics assay showed a progressive inactivation of all tested bacterial strains after their exposure to EO for 8 h at MICs. Furthermore, Molecular docking showed remarkable affinities between EO components, target proteins and DNA gyrase subunit B (GyrB). Moreover, the in silico ADMET predictions provided preliminary insights into the safety-related properties of the major EO components. In addition, EO compounds have the potential to interact with bacterial structures. These findings highlight the in vitro antibacterial potential of the *M. rotundifolia* EO and suggest its promise as a natural source of bioactive compounds.

## 1. Introduction

Throughout antiquity, medicinal and aromatic plants have been recognized by all civilizations and cultures as a significant source of therapeutic remedies due to their biological activities [1]. However, in recent times, they have gained greater attention driven by heightened consumer awareness of synthetic food preservatives [2,3], which were aimed at extending the shelf-life of food and are extensively utilized because of their low cost and high efficiency. These include nitrate, nitrite, sodium, potassium and sorbate used in food processing [4]. In response to public concerns, previous studies have already confirmed that certain chemicals may pose a risk to human health and that some of them may, over the long term, lead to chronic diseases (e.g., cancer) due to the accumulation and migration of chemical residues throughout the food preservation process [5,6]. To this end, researchers have increasingly focused on the use of aromatic and medicinal plants as a promising alternative to synthetic preservatives [7].

In this context, considerable interest has gradually focused on the Lamiaceae family, which includes several well-known aromatic plants [8]. This family comprises approximately 7200 plant species [9], recognized for their culinary properties, as seasonings and flavoring agents, as well as for their biological and medical applications [10,11]. Among the most investigated representatives of this family is the *Mentha* genus, recognized for its economic value [12]. In fact, *Mentha* species, well known for their many traditional applications and uses, have been the subject of extensive research, particularly for their antibacterial properties [13], probably due to the richness and diversity of the bioactive components of their essential oil (EO). Previous studies conducted on several species of the genus have revealed considerable variability in their chemical profiles and associated biological activities [14,15]. Among these species, *M. rotundifolia* is a notable example, distinguished by its chemical composition and the significant presence of Rotundifolone, the proportion of which varies based on geographical origin.

Although the EO of *M. rotundifolia* has been explored in several earlier studies [16,17], the data reported in the literature remain heterogenous and essentially descriptive. Existing studies are mostly limited to identifying the chemical composition and evaluating in vitro antibacterial activity without establishing any in-depth link between the major compounds, their molecular proprieties, and the mechanisms likely to underlie the observed activity.

In this context, to overcome the limitations in the exploration of the antibacterial mechanisms of *M. rotundifolia* EO, an integrated multi-step approach was adopted. First, GC-MS/MS analysis was used to identify the major chemical constituents of the EO extracted from the plant’s leaves. These compounds were then subjected to DFT (Density Functional Theory) calculations to evaluate their electronic properties and chemical reactivity, which are key factors influencing their biological interactions. Subsequently, molecular docking was performed to predict the binding affinity of the selected compounds with relevant bacterial targets, providing insights into their possible mechanism of action. Finally, ADMET (Absorption, Distribution, Metabolism, Excretion, and Toxicity) prediction was conducted to assess the pharmacokinetic behavior and safety profiles of the most active EO components. This combined workflow enables a deeper mechanistic interpretation linking chemical composition to antibacterial activity.

The results obtained therefore provide a preliminary scientific basis for cautiously exploring the use of *M. rotundifolia* EO in potential food applications, subject to further investigations studies tailored to real food systems.

## 2. Results and Discussion

### 2.1. Determination of Organoleptic Properties

Since the EO consists of natural components that can be affected or destroyed by various external factors, assessing its organoleptic properties is crucial, as this provides a better understanding of its physicochemical characteristics and quality [18]. The assessment began with the examination of the first parameter, the smell, which had a strong odor suggesting characteristics of a mixture of mint and thyme. This strong smell has been recently well-documented in the same species [19]. Regarding appearance and color, it appeared pale yellow and displayed a clear liquid quality, devoid of turbidity or deposits (Figure S1).

### 2.2. Determination of Physical Properties

Determining physical properties of an EO allows for the assessment of its purity and quality and provides crucial information for the development of new products, given the wide range of its potential applications. The analysis of parameters measured at ambient temperature showed that the analyzed EO had a freezing point of −20 °C. This result determines the storage and usage conditions of EO in pharmaceutical, cosmetic and/or food applications. According to a previous study, the freezing point of *M. suaveolens* EO was found to be −10 °C [19]. These findings suggest that each EO has a specific freezing point depending on the investigated species and its components.

Regarding the other parameters, analyses showed that the tested EO exhibited remarkable miscibility with ethanol; 1 volume of EO mixed with 1 volume of ethanol produces a uniform mixture without separation of the two phases. These findings are consistent with a previous study, which reported an equivalent volume of ethyl alcohol for EO of *Eucalyptus globulus* [20]. The third parameter studied is relative density, defined as the mass of the EO per unit volume compared to that of water at the same temperature. This measurement provides insight into the composition and physical characteristics of the oil, as variations in molecular structure and molecular weight can affect density values [20]. The analysis yielded a relative density of 0.7791, which is very close to the value recently reported for EO of *Mentha arvensis* [20]. In contrast, recent studies have reported higher relative density values of 1.456 and 1.0350 for the EO of *Eucalyptus globulus* and *Syzygium aromaticum*, respectively [20].

### 2.3. Chemical Profile of EO

The chemical profile of the EO extracted from *M. rotundifolia* leaves collected from Central-Southern Morocco focused on determining its chemical components using GC-MS/MS. Results revealed that the general composition of the EO consisted mainly of oxygenated monoterpenes (72.45%), followed by sesquiterpenes (16.7%), and non-oxygenated monoterpenes of 4.95%, and that a total of 27 components were present in the analyzed EO, representing 96.19% of the total compounds. The major chemicals identified in the investigated EO are summarized in Table 1. As shown, the composition is dominated by Rotundifolone, Carvacrol, Piperitenone, and Pulegone, with percentages of 27.95%, 19.48%, 6.09%, and 4.47%, respectively.

Comparable results were obtained in Tunisia regarding the components of EO from *M. rotundifolia* sampled in two distinct regions, each with a different profile [21]. The authors showed that in the first region, *Bizerte*, the EO is primarily dominated by oxygenated monoterpenes, with the presence of Pulegone (32.09%) and Piperitenone oxide (17.28%), while in the second region, *Beja*, sesquiterpenes were predominant. Conversely, this contrasts with the chemical profile from EO of *M. rotundifolia* collected in Algeria, which revealed the detection of almost 77 components, accounting for 98.19% of the total EO composition, with the predominance of [Cyclobutaneacetonitrile,1-methyl-2-(1-methylethenyl)-], terpinene-4-ol, p-menthane, germacrene, myrcene, and caryophyllene, with 45.60, 6.36, 5.46, 4.49, 3.04, and 2.29%, respectively [22]. A study conducted in Iran revealed that monoterpenes and sesquiterpenes dominated the chemical components identified in *M. spicata* EO, with Carvone, Limonene, β-elemene, and Caryophyllene being the most prevalent constituents [23]. In addition, other studies have reviewed the chemical profile of EO obtained from the leaves of numerous *Mentha* species, such as *M. piperita*, *M. longifolia*, *M. pulegium*, *M. arvensis*, *M. spicata*, *M. suaveolens*, *M. arvensis*, *M. aquatica*, and *M. viridis*, and have emphasized that, in general, monoterpenes and sesquiterpenes dominate the chemical components identified in the analyzed EO, suggesting that this chemical class could be an intrinsic characteristic or a chemical fingerprint of *Mentha* species [24,25].

### 2.4. Antibacterial Activity

**Figure 1:**
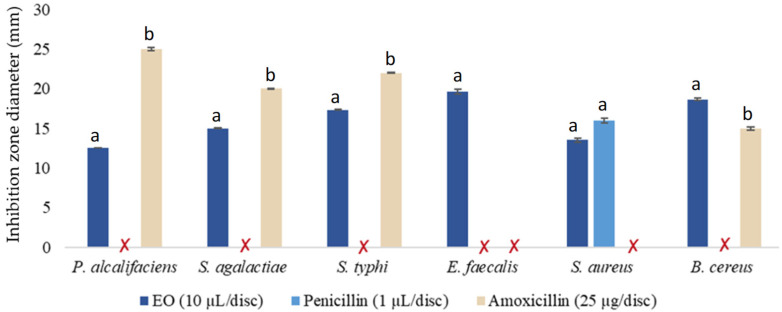
Diameters of inhibition zones (mm) obtained following the effect of *M. rotundifolia* EO on the tested bacterial strains. (Note: Values are presented as mean ± standard deviation (SD), n = 3. Bars with different letters indicate significant differences (*p* < 0.05) according to one-way ANOVA followed by Tukey’s post hoc test.)

The antibacterial activity of *M. rotundifolia* EO was evaluated using the disk diffusion method, considered a qualitative screening tool. The results revealed that the EO induced inhibition zones with diameters that varied depending on the tested bacterial strains reflecting differentiated sensitivity. Indeed, *E. faecalis* CIP 103214 showed the maximum clearance zone in response to the tested EO, with an inhibition diameter of 19.66 mm, while this strain has been shown to be resistant to Amoxicillin and Penicillin, two antibiotics used as positive controls for the purpose of evaluating the observed antibacterial activity. However, any direct comparison with the reference antibiotics remains limited given the differences in chemical nature of mechanisms of action, concentration units, and diffusion in the agar medium. Furthermore, a remarkable antibacterial effect was also observed against *B. cereus* ATCC 33019 with an inhibition zone diameter of 18.66 mm. In contrast, the lowest inhibition zone (12.55 mm) was noted *versus P. alcalifaciens* CIP82.90T (Figure 1).

Earlier records from Maghreb countries (*Algeria*, *Morocco*, and *Tunisia*) indicated that EO of *M. rotundifolia* exhibited excellent activity against various bacterial strains, as summarized in Table 2. As shown, the EO of *M. rotundifolia* from Morocco achieved a remarkable inhibition zone diameter of 56 mm against *Proteus mirabilis* [26]. However, according to an Algerian study, *M. rotundifolia* EO showed no activity against *Streptococcus pyogenes CIPA22* and *Pseudomonas aeruginosa CIPA22* [27]. 

**Figure 2:**
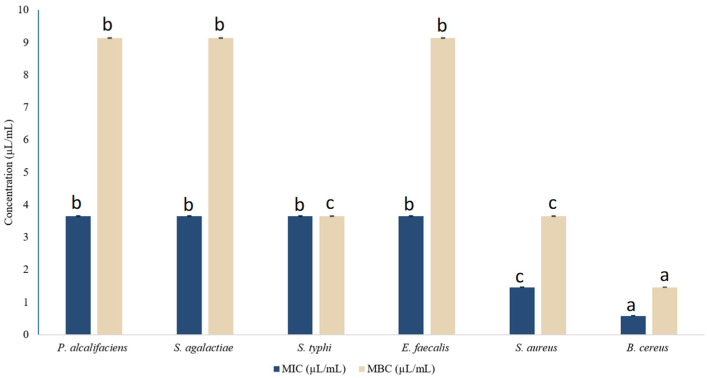
MIC and MBC values of *M. rotundifolia* EO tested against the targeted bacterial strains. (Note: Values are presented as mean ± standard deviation (SD), n = 3. Bars with different letters indicate significant differences (*p* < 0.05) according to one-way ANOVA followed by Tukey’s post hoc test).

In addition to the disk diffusion method, a quantitative assessment was performed by determining the minimum inhibitory concentration (MIC) and minimum bactericidal concentration (MBC). Results represented in Figure 2 indicated that MIC values ranged from 0.58 to 3.65 µL/mL, while MBC values varied from 1.46 to 9.14 µL/mL. Among the tested strains, *B. cereus* ATCC 33019 showed the highest sensitivity, with MIC and MBC values of 0.58 and 1.46 µL/mL, respectively. For comparison, the MIC and MBC values obtained with the positive control (streptomycin) in this test were 30 ± 0.01 and 90 ± 0.01 µg/mL, respectively. Although DMSO can affect bacterial growth as stated earlier, the concentration used in this study (5%) showed no inhibitory effect, indicating that *M. rotundifolia* EO exerted a remarkable inhibitory effect against *B. cereus* ATCC 33019. These findings showed significant antibacterial efficacy of *M. rotundifolia* EO compared to previously reported effect on *S. aureus* growth, with MIC value of 18 ± 0.41 mg/mL [33]. On the other hand, a lower MIC value (0.48 mg/mL) has been reported for *M. suaveolens* EO against *S. aureus*, suggesting a comparatively higher antibacterial activity. Such differences are commonly observed among *Mentha* species and can be attributed to a variety of factors, including differences in extraction methods, variations in chemical composition, geographic origin, and methodological conditions [19].

It should be noted that many studies have reported that the chemical composition of EO affects its bioactivity, particularly its antibacterial action [34]. As mentioned above, the EO of *M. rotundifolia* is composed of both oxygenated monoterpenes (72.45%) and sesquiterpenes (16.7%), and it has been reported that this class of chemicals (monoterpenes) exhibits good antibacterial activity [34]. Their hydrophobicity, a key characteristic, enables them to partition into the lipids of the bacterial cell membrane, disrupting its structure and increasing its permeability [35]. This can result in leakage of ions and a greater loss of cell contents [36], or in the critical output of molecules and ions that can cause cell death [37].

### 2.5. Time-Kill Kinetics

**Figure 3:**
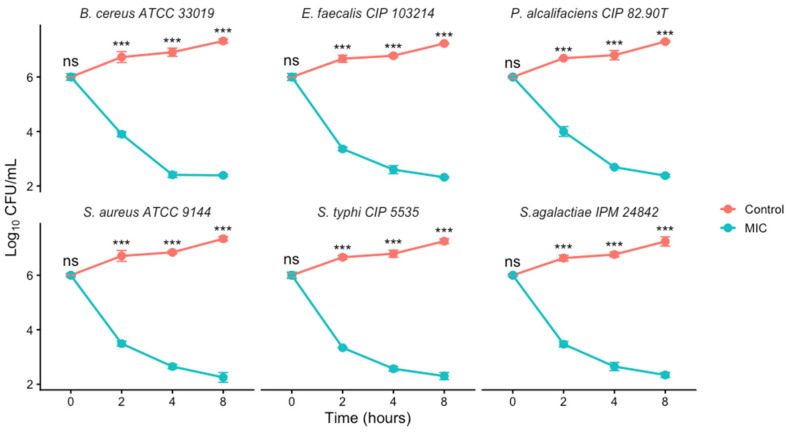
Time-kill kinetics of *M. rotundifolia* EO against the tested bacterial strains. (Note: Values are presented as mean ± standard deviation (SD), n = 3. Statistical significance was determined by one-way ANOVA followed by Tukey’s post hoc test. Significance is indicated as follows: *** *p* < 0.001, ns = not significant.)

The time-kill assay was performed in this study using an initial inoculum of approximately 1 × 10^6^ CFU/mL to assess the antibacterial activity of *M. rotundifolia* EO at MICs over 8h time against each bacterial strains. As illustrated in Figure 3, the time-kill curves are presented in six individual panels, each corresponding to a bacterial strain. Overall, exposure of bacterial strains to *M. rotundifolia* EO at the MIC concentration resulted in a time depending decrease in viable cell counts across the tested strains. For instance, in *S. aureus* ATCC 9144, the viable count decreased from approximately 6.0 log_10_ CFU/mL at baseline to 2.25 log_10_ CFU/mL after 8 h of exposure. In contrast, the untreated control with *P. alcalifaciens* CIP 82.90T showed continuous bacterial growth, increasing from the initial inoculum to 7.3 log_10_ CFU/mL after 8 h of growth, and similar trends were observed for the remaining strains. The time-dependent behavior observed in this study aligns with previous reports describing progressive bacterial inactivation following exposure to plant-derived EO [38,39]. This kinetic pattern is commonly associated with membrane disruption, increased permeability, and leakage of intracellular constituents caused by the hydrophobic components of EO [38]. Overall, the finding support the potential of *M. rotundifolia* EO as an effective antimicrobial agent, exhibiting reproducible activity across multiple bacterial strains.

**Figure 4:**
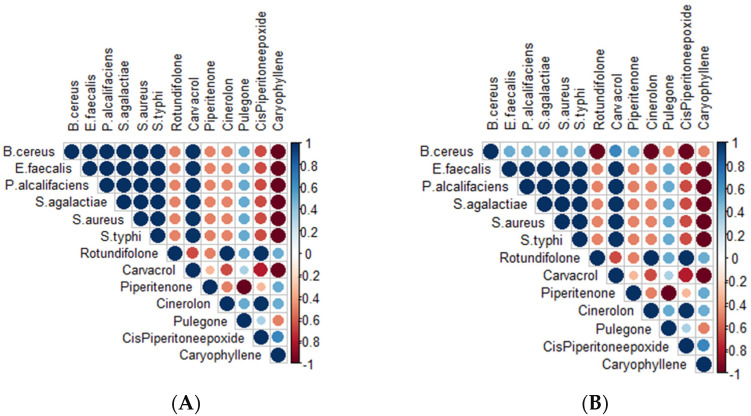
(**A**): Correlation of major chemical compounds identified in the EO with MIC values. (**B**): Correlation of major chemical compounds identified in the EO with MBC values. (Note: The color gradient reflects both the direction and strength of the correlation: increasing intensity toward dark brown indicates a stronger negative correlation. Circle size represents the magnitude of the correlation coefficient.)

To further elucidate the potential relationship between components of *M. rotundifolia* EO listed in Table 1 and the measured antibacterial parameters, a correlation analysis was preformed between the relative abundances of EO major components and the MIC and MBC values obtained in this study. Findings shown in Figure 4 revealed distinct association profiles depending on the studied compounds. To illustrate, Caryophyllene compound showed a negative correlation with MIC and MBC values suggesting a potential association with increased antibacterial activity. Similarly, Cis-piperitone epoxide, Rotundifolone, Cinerolon and Piperitenone all exhibited a comparable trend, although the intensity of the correlation was moderate. In contrast, Pulegone and Carvacrol indicated a positive correlation with the antibacterial activity’s results, indicating limited or indirect involvement in the observed activity.

The correlation analysis has uncovered an association between oxygenated monoterpenes and MIC and MBC values supporting the potential involvement of this chemical class in the observed antibacterial activity (Table S1). These findings fully aligned with those of a previous study on *M. rotundifolia* EO from Tunisia [40]. Conversely, a previous study from Morocco attributed the antibacterial activity to the predominance of phenols in the EO of endemic thymes against multi-resistant bacteria [41]. Nevertheless, this association remains to be explored because, so far, no direct relationship has been previously established. Thus, further research on isolated compounds from EO is therefore needed.

It should be noted that the antibacterial mechanisms proposed in this study, including membrane disruption and enzyme inhibition, are based on previously reported literature data and supported by the observed in silico interactions. However, these mechanisms have not been experimentally validated in the present work. Therefore, they should be considered as plausible hypotheses rather than confirmed modes of action. Thus, further experimental investigations, such as membrane integrity assays or enzymatic studies, are required to validate these proposed mechanisms and to better understand the mode of action of the investigated EO and its chemical components.

### 2.6. DFT and MESP Analysis

**Figure 5:**
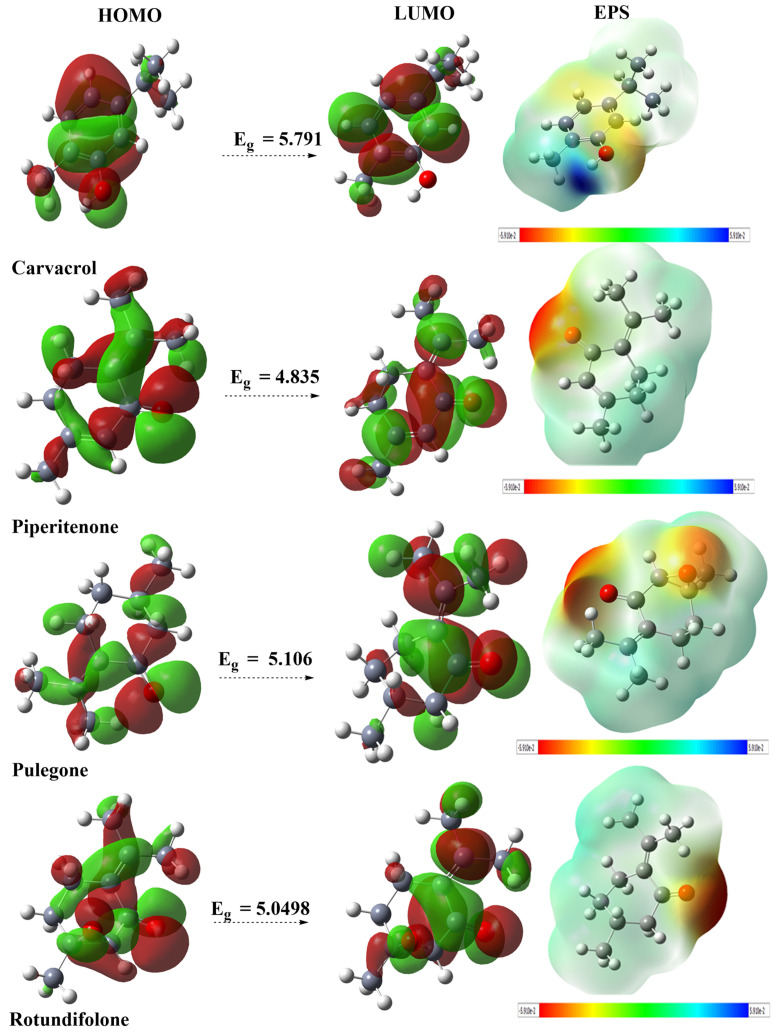
Diagrams of the highest occupied molecular orbital (HOMO), lowest unoccupied molecular orbital (LUMO) and corresponding band gap, as well as electrostatic potential (ESP) maps of the main compounds identified in the EO, are shown. All calculations were performed at the B3LYP/6-31G+(d,p) theoretical level.

All optimized structures correspond to minima on the potential energy surface (characterized by positive vibrational frequencies). The total energies obtained for the studied compounds shown in Figure 5 allowed the classification of their stability as follows: Rotundifolone > Pulegone > Carvacrol > Piperitenone. Additionally, other parameters such as the energies of the frontier orbitals (HOMO and LUMO), as well as descriptors of chemical reactivity, are presented in Table 3. DFT descriptors provide information on the intrinsic electronic structure and theoretical chemical reactivity of the studied compounds. However, they do not allow for a direct prediction of biological activity. In this study, their role is to complement the structural and docking analyses by providing a theoretical description of the electronic regions likely to interact.

#### 2.6.1. Frontier Molecular Orbitals (FMOs)

In the context of discovering new bioactive compounds, DFT calculations can be used to characterize the fundamental electronic properties of molecules. These properties, such as the energies of the highest occupied molecular orbitals (HOMOs) and lowest unoccupied molecular orbitals (LUMOs) and the Energy Gap (Eg), provide information on the theoretical chemical reactivity and the stability of compounds [42]. In this study, DFT was used to establish a profile of these electronic properties for the main EO components, thus complementing the structural analysis obtained from molecular docking. Data in Table 3 summarize these energy values. The FMO-derived descriptors were used to characterize the intrinsic electronic features of the compounds, whereas the docking results are mainly governed by local non-covalent interactions within the protein binding pocket. Thus, the electrophilic/nucleophilic analysis should be viewed as complementary theoretical information that may help to rationalize, but not directly to predict, protein-ligand binding. Figure 4 shows that the Eg gap allows the compounds to be classified according to their electronic stability: Carvacrol > Pulegone > Rotundifolone > Piperitenone. This suggests that carvacrol may have higher theoretical chemical reactivity, while its electronic structure indicates relatively lower kinetic stability compared to other compounds.

The energy values allow calculation of several chemical reactivity indices, including chemical potential (µ), which provides insight into a molecule’s tendency to donate or accept electrons [43]. Examination of data in Table 3 shows that µ decreases in the following order: Carvacrol > Pulegone > Piperitenone > Rotundifolone, which suggests that rotundifolone may be more likely to participate in electronic interactions. A higher dipole moment could promote polar interactions with amino acid residues within the binding site, which could influence the interaction patterns observed in molecular docking simulations.

The theoretical chemical reactivity of EO components was characterized using global electronic descriptors, notably electrophilicity and nucleophilicity indices. Electrophilic substances target electron-rich sites, while nucleophilic substances interact with electron-deficient sites. Data shown in Table 3 indicate that all the studied chemicals exhibited significant nucleophilic character, with decreasing strength in the following order: Carvacrol > Piperitenone > Pulegone > Rotundifolone [44]. In contrast, the electrophilic indices showed that Rotundifolone and Piperitenone are strong electrophiles, while Pulegone and Carvacrol are more moderate [45].

This dual profile, combined with parameters such as hardness and chemical potential, suggests diverse intrinsic electronic reactivity within the EO. This diversity of electronic descriptors can influence the way molecules interact with protein binding sites, as observed in molecular docking simulations. For example, the less electrophilic profiles of Carvacrol and Pulegone could correspond to complementary modes of interaction with protein active sites, which may contribute to differences in interaction modes with protein active sites.

#### 2.6.2. Analysis Electron Localization Function (ELF)

**Figure 6:**
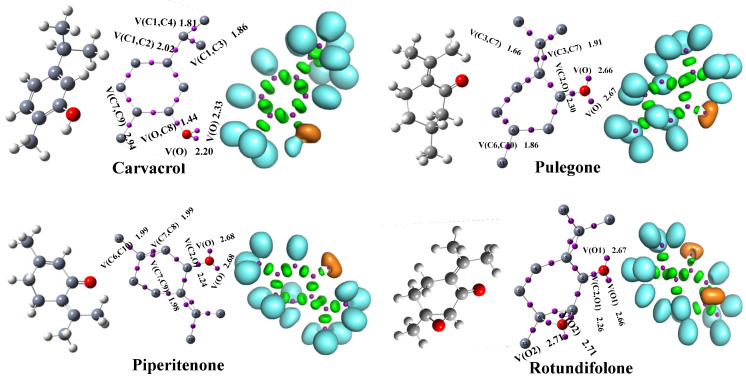
Electron Localization Function (ELF) maps of Carvacrol, Pulegone, Piperitenone, and Rotundifolone computed at the B3LYP/6-31G+(d,p) level.

Electron localization function (ELF) analysis provides a visual description of the spatial distribution of electrons within the studied molecules. The ELF maps of Pulegone, Rotundifolone, Piperitenone, and Carvacrol (Figure 6) revealed highly localized regions (cyan) mainly around the oxygen atoms of the carbonyl and hydroxyl groups, corresponding to lone pair electron domains and polar covalent bonds. These regions highlight potential sites of electronic interaction that may participate in intermolecular interactions. The green areas observed above the carbon rings indicate partial π-electron delocalization, reflecting the electronic distribution within the aromatic or unsaturated frameworks of the molecules. Such delocalization contributes to the internal electronic organization of the structures. The results also indicate that Rotundifolone and Carvacrol exhibit a relatively more homogeneous electron density distribution, while Pulegone and Piperitenone present more localized electron density regions around specific functional groups. These electronic features provide insight into the intrinsic electronic structure of the studied molecules and may influence their interaction patterns with biological targets observed in molecular docking simulations. Overall, the ELF analysis highlights the role of oxygen-containing functional groups in shaping the electronic environment of these compounds, which may contribute to their potential intermolecular interactions.

#### 2.6.3. Analysis of Non-Covalent Interactions (NCI)

**Figure 7:**
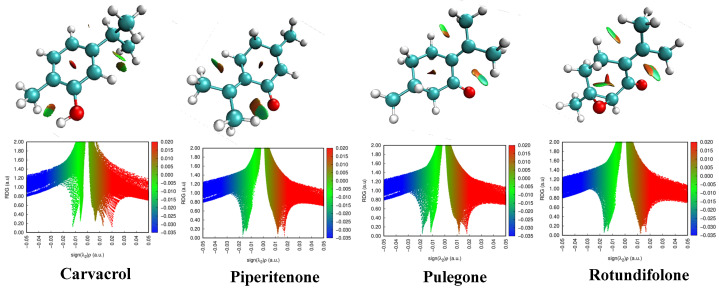
Reduced density gradient (RDG) plots as a function of ρ × sign (λ_2_) and colored 3D RDG isosurfaces for Carvacrol, Pulegone, Piperitenone, and Rotundifolone computed at the B3LYP/6-31G+(d,p) level.

The non-covalent interaction index (NCI) is a powerful theoretical tool that enables the visualization and identification of weak molecular interactions, whether attractive (hydrogen bonds, π–π interactions) or repulsive (steric effects) [46,47]. Based on reduced density gradient (RDG) analysis, the NCI method provides a qualitative and intuitive description of the spatial regions where non-covalent forces occur, which play a crucial role in molecular stability, reactivity and structural organization. These interactions play an important role in the structural organization and intermolecular interactions of many molecular systems [48]. NCI analysis of Pulegone, Rotundifolone, Piperitenone and Carvacrol (Figure 7) revealed the presence of non-covalent interactions contributing to their conformational stability. The green regions indicate the predominance of *van der Waals* interactions, which are responsible for internal stabilization without the formation of strong covalent bonds. The blue areas, more pronounced in Pulegone and Rotundifolone, correspond to attractive hydrogen bond-type interactions, which suggests stronger and more attractive intramolecular interactions. In the case of Carvacrol, the presence of a hydroxyl group promotes intramolecular hydrogen bonds, which could affect its electronic distribution and molecular structure. Thus, this analysis provides a detailed map of the weak interactions that underlie the conformational stability of these molecules and could lead to a better understanding of the electronic properties that may influence intermolecular interactions. 

#### 2.6.4. MEP Analysis of Compounds

Examining charge distribution, and investigating hydrogen bond interactions [49], MEP analysis highlights electropositive regions (blue), which support nucleophilic attacks, and electronegative regions (red), which are susceptible to electrophilic attacks [50]. MEP map analysis (Figure 6) revealed that the oxygen atom of the C=O groups in Pulegone, Rotundifolone, and Piperitenone is a sensitive site for electrophilic attacks (red regions), while the OH group of Carvacrol acts as a nucleophilic site, the blue area around the hydrogen of the hydroxyl group reflects the strong polarization of the O-H bond. This polarization results from a strong attraction of electrons by oxygen. This makes the hydroxyl group a very important functional group in hydrogen bonding interactions. These electrostatic properties can influence the ability of molecules to participate in intermolecular interactions such as hydrogen bonding [51]. The theoretical calculations (DFT, MESP, ELF, NCI) were included to characterize the electronic structure of the main compounds and to identify interaction-prone regions that may contribute to intermolecular recognition. These results should be regarded as complementary to the experimental and docking data, not as direct evidence of antibacterial mechanism.

#### 2.6.5. Molecular Docking

##### Choice of Molecular Target

The selection of target proteins for molecular docking screening was based on their essential role in the physiology or virulence of bacteria that play an important role in food safety and food spoilage [52,53]. For *B. cereus*, lactate dehydrogenase (3WT0) was chosen because of its central role in anaerobic energy metabolism, a condition found in many foods [54]. Gyrase B from *S.* Typhi (6J90) and *E. faecalis* (4M7U), an enzyme essential for DNA replication, represents a classic bactericidal target for evaluating broad-spectrum potential. Against *S. aureus*, we targeted thermonuclease (5CDP), a key virulence factor involved in food poisoning [55,56,57]. The *P. alcalifaciens* transporter (6LK2) was selected to interfere with nutrient absorption at low temperatures [58]. In addition, the *S. agalactiae* LTA-binding protein (5Y2G) was included as a model structural target for Gram-positive bacteria, relevant in the context of milk contamination [59]. Finally, the DNA gyrase subunit B (GyrB), (7P2N) was selected to be tested with EO major components. The choice of GyrB was motivated by its satisfactory crystallographic resolution and its crucial role in bacterial DNA replication. In fact, inhibition of GyrB blocks cell division, making it a target of choice for assessing the antibacterial potential of compounds destined to biological applications and food preservation [60,61].

##### Docking Validation Based on Ligand Pose Superposition

**Figure 8:**
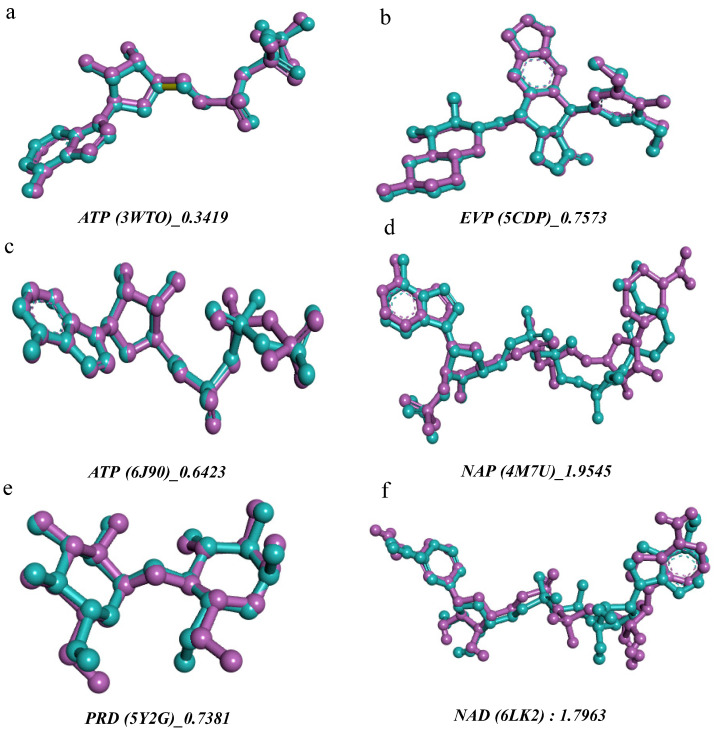
Superimposition of experimentally determined co-crystallized ligands (purple) and their corresponding redocked poses (cyan) for different protein structures. RMSD values (Å) are reported as a geometric indicator of pose similarity. Note: (**a**): APT for *B. cereus* (PDB ID:3WT0), (**b**): EVP for *S. aureus* (PDB ID: 5CDP), (**c**): ATP for *S.* Typhi (PDB ID: 6J90), (**d**): NAP for *E. faecalis* (PDB ID: 4M7U), (**e**): PRD for *S. agalactiae* (PDB ID: 5Y2G), and (**f**): NAD for *P. alcalifaciens* (PDB ID: 6LK2).

The reliability of the docking protocol was validated by redocking the native ligands to their respective crystallographic complexes (Figure 8), a: APT for *B. cereus* (PDB ID:3WT0), b: EVP for *S. aureus* (PDB ID: 5CDP), c: ATP for *S.* Typhi (PDB ID: 6J90), d: NAP for *E. faecalis* (PDB ID: 4M7U), e: PRD for *S. agalactiae* (PDB ID: 5Y2G), f: NAD for *P. alcalifaciens* (PDB ID: 6LK2), and g: 4R3 for the DNA gyrase subunit B (GyrB) (PDB ID: 7P2N). The low root means square deviations (RMSD) obtained, ranging from 0.34 to 1.95 Å, attest to the excellent reproduction of the experimental conformations and validate the robustness of the parameters used for the main simulations.

In silico modeling by molecular docking of the main compounds of *M. rotundifolia* EO (Rotundifolone, Pulegone, Piperitenone, and Carvacrol) to target proteins of six bacterial strains (*B. cereus*, *E. faecalis*, *S.* Typhi, *S. aureus*, *S. agalactiae*, and *P. alcalifaciens*) and the DNA gyrase subunit B (GyrB, PDB: 7P2N) revealed binding energies ranging from −5.4 to −7.2 kcal/mol (Table 4). Streptomycin (−5.7 to −8.7 kcal/mol) was included as a reference ligand because it was used as an experimental antibacterial control. However, the comparison of docking scores with small monoterpenes should be interpreted with caution due to differences in molecular size and structural complexity.

Relatively favorable binding scores were predicted for Carvacrol towards *B. cereus* and GyrB, as well as for Piperitenone and Rotundifolone towards *E. faecalis* and *S. aureus*. The binding configurations suggest the presence of hydrogen bonds and hydrophobic contacts (via carbonyl and hydroxyl groups), as well as hydrophobic interactions and *Van der Waals* forces with residues in the active sites, which could contribute to the predicted binding mode of the protein-ligand complexes.

Furthermore, a certain degree of consistency was observed between the electronic descriptors and the interaction modes obtained by docking. Nevertheless, this association should be interpreted with caution, as chemical reactivity descriptors do not allow for the direct validation of docking predictions or biological activity. The docking results thus suggest a possible interaction between certain EO components and selected bacterial protein targets, including DNA gyrase B. However, these results should be considered as in silico predictions of potential affinity and not as confirmed evidence of enzymatic inhibition. Indeed, the antibacterial activity of the EO is likely multifactorial and may involve membrane disruption, altered permeability, leakage of intracellular contents, as well as potential interactions with intracellular targets [62,63,64].

On the other hand, molecular docking analysis provides useful insights into the potential interactions between the studied compounds and selected biological targets. However, it is important to emphasize that docking-derived binding energies reflect only in silico affinity and cannot be directly interpreted as a measure of antibacterial efficacy. This limitation is particularly relevant when comparing structurally simple monoterpenes with a complex reference antibiotic. Therefore, the docking results presented in this study should be considered as indicative of possible interaction modes rather than definitive evidence of biological activity. The observed differences in antibacterial performance are likely influenced by additional factors such as membrane permeability, compound solubility, and metabolic stability.

A comparative in silico analysis including non-oxygenated monoterpenes would be valuable to further evaluate the contribution of oxygenated functional groups to target recognition and antibacterial potential. This aspect is considered a relevant perspective for future investigations. This multiplicity of effects represents a strategic advantage for limiting the development of bacterial resistance, and the positioning of EO of *M. rotundifolia* as a promising natural candidate for biological food preservation, in response to the growing demand for natural substances to limit the use of synthetic chemical additives.

**Figure 9:**
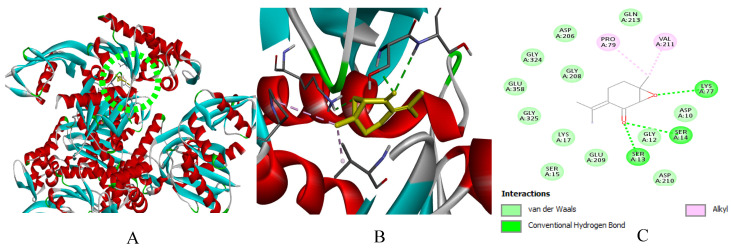
(**A**): Ligand positioning in the active site of *B. cereus* (PDB ID: 3WT0), (**B**): Three-dimensional representation of the interactions between Rotundifolone and the active site of *B. cereus*, and (**C**): Two-dimensional diagram illustrating the interactions of amino acid residues within the binding pockets. The different colors of the residues reflect the nature of the interactions.

**Figure 10:**
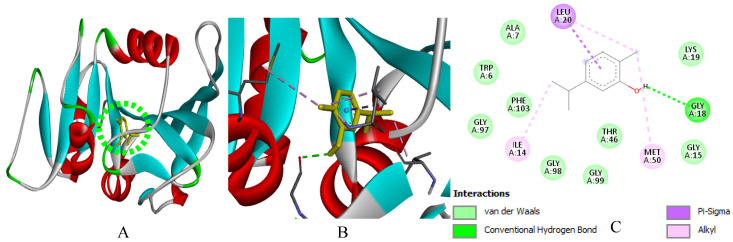
(**A**): Ligand positioning in the active site of *E. faecalis* (PDB ID: 4M7U), (**B**): Three-dimensional representation of the interactions between Carvacrol and the active site of *E. faecalis*, and (**C**): Two-dimensional diagram illustrating the interactions of amino acid residues within the binding pockets. The different colors of the residues reflect the nature of the interactions.

**Figure 11:**
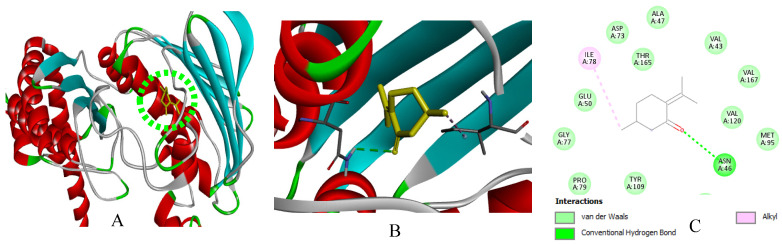
(**A**): Ligand positioning in the active site of *S.* Typhi (PDB ID: 6J90), (**B**): Three-dimensional representation of the interactions between Pulegone and the active site of *S.* Typhi, and (**C**): Two-dimensional diagram illustrating the interactions of amino acid residues within the binding pockets. The different colors of the residues reflect the nature of the interactions.

**Figure 12:**
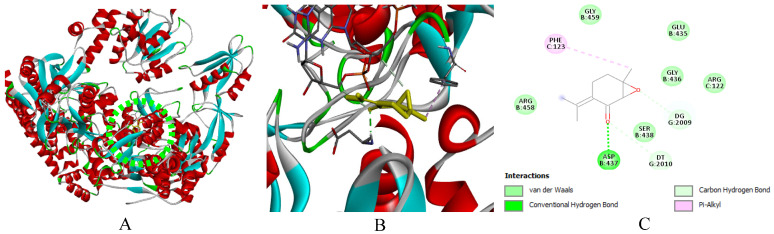
(**A**): Ligand positioning in the active site of *S. aureus* (PDB ID: 5cdp), (**B**): Three-dimensional representation of the interactions between Rotundifolone and the active site of *S. aureus*, and (**C**): Two-dimensional diagram illustrating the interactions of amino acid residues within the binding pockets. The different colors of the residues reflect the nature of the interactions.

**Figure 13:**
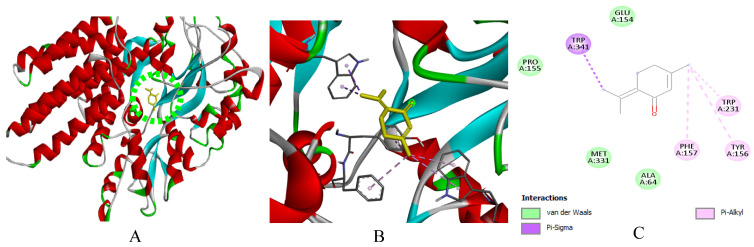
(**A**): Ligand positioning in the active site of *S. agalactiae* (PDB ID: 5y2g), (**B**): Three-dimensional representation of the interactions between Piperitenone and the active site of *S. agalactiae*, and (**C**): Two-dimensional diagram illustrating the interactions of amino acid residues within the binding pockets. The different colors of the residues reflect the nature of the interactions.

**Figure 14:**
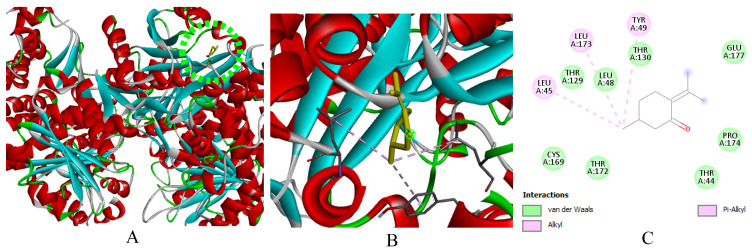
(**A**): Ligand positioning in the active site of *P. alcalifaciens* (PDB ID: 6lk2), (**B**): Three-dimensional representation of the interactions between Pulegone and the active site of *P. alcalifaciens*, and (**C**): Two-dimensional diagram illustrating the interactions of amino acid residues within the binding pockets. The distinct colors of the residues reflect the nature of the interactions.

**Figure 15:**
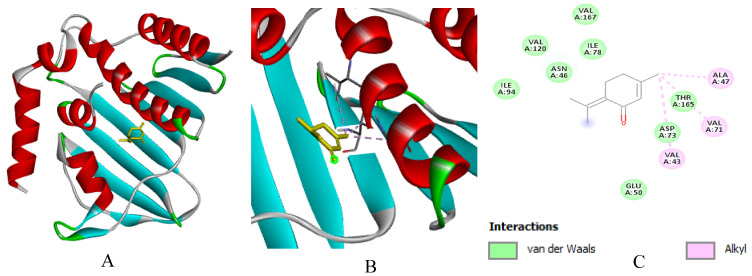
(**A**): Ligand positioning in the active site of GyrB (PDB ID: 7P2N), (**B**): Three-dimensional representation of the interactions between piperitenone and the active site of GyrB, and (**C**): Two-dimensional diagram illustrating the interactions of amino acid residues within the binding pockets. The distinct colors of the residues reflect the nature of the interactions.

Due to their significant proportion and efficacy comparable to that of Streptomycin, the findings of this study suggest that Piperitenone and Rotundifolone are fundamental to the biological activity of the studied EO of *M. rotundifolia*. Furthermore, the accuracy of the predictions is confirmed by the strong association between chemical reactivity and docking results. Figure 9, Figure 10, Figure 11, Figure 12, Figure 13, Figure 14 and Figure 15 show the interaction patterns of the optimal ligand with each studied target.

Finally, it should be highlighted from results of the present study that the antibacterial activity observed following MIC and MBC determination showed a consistent qualitative agreement with the in silico predictions. Indeed, compounds exhibiting favorable binding affinities and stable interactions with key amino acid residues of the target proteins are likely to contribute to the observed antibacterial effects. This concordance suggests that the proposed molecular targets may be relevant to the mechanism of action of the tested EO components. However, it is also important to note that this relationship remains primarily qualitative rather than strictly quantitative. Indeed, the biological activity of EO constituents may be influenced by multiple factors, including membrane permeability, compound stability, bioavailability, and potential synergistic interactions between components. These parameters are not fully captured by molecular docking approaches. Consequently, although the in silico results support the experimental findings and provide a mechanistic rationale for them, they should be interpreted with caution and viewed as supplementary evidence rather than as definitive proof of the observed antibacterial activity.

#### 2.6.6. In Silico Assessment of the Toxicological Profile

The safety evaluation of the main components of *M. rotundifolia* EO for biopreservative applications can be initially approached through an in silico toxicity prediction, which provides a valuable preliminary screening tool. In silico toxicological analysis of the major identified compounds (Carvacrol, Piperitenone, Pulegone, Rotundifolone) revealed no mutagenic risk (AMES test) or hepatotoxicity (Table 5). Their acute (LD50) and chronic (LOAEL) toxicity is moderate and similar between molecules. However, all present a risk of skin sensitization, requiring precautions during handling. Their environmental toxicity varies depending on the compound and the target organism (Rotundifolone is less toxic to Tetrahymena pyriformis).

In general, the predicted toxicological profiles of the investigated compounds suggest minor toxicity, supporting their potential use in controlling microbial growth in food systems. However, these predictions remain theoretical and should be interpreted with caution, as they do not fully account for physiological complexity, matrix interactions, or long-term exposure effects. Furthermore, such predictions are constrained by the inherent limitations of in silico models, which cannot fully replicate the complexity of biological systems, including matrix effects and dynamic physiological environments.

## 3. Materials and Methods

### 3.1. Plant Material

The studied plant, *M. rotundifolia* (L.) Huds, was collected during the flowering period in 2024 in the Central-Southern area of Morocco, and its taxonomy was confirmed by the National Agency for Aromatic and Medicinal Plants (ANPMA). The sampling area is classified as having high-temperature, with an arid climate [65]. It is characterized by an annual rainfall of 208 mm (P) and temperatures ranging from a minimum of 19 °C in January to a maximum of 37 °C in July.

### 3.2. EO Extraction

After sorting and purification of the plant, the leaves were dried at ambient temperature in the shade for 15 days, then prepared for hydro-distillation using a Clevenger apparatus for 3 h, at a ratio of 100 g of dry plant materiel in 1 L of distilled water. The EO was obtained with a yield of 0.33%, then stored in a dark vial at 4 °C until further analysis.

### 3.3. Organoleptic Properties

The organoleptic properties of *M. rotundifolia* EO were assessed by examining the color, viscosity, clarity, and odor of the oil following its recovery, according to the protocol of AFNOR, as previously described [19].

### 3.4. Physical Properties

#### 3.4.1. Relative Density

This test was performed according to the protocol of AFNOR standards for EO analysis [19]. Briefly, a 1 mL Eppendorf tube, tared to zero on the balance, was used to calculate the relative density. The tube was then weighed after being filled with 200 µL of the tested EO, and the same process was repeated for distilled water. The following formula was applied to determine the relative density:

(1) $$Relative\;density=\frac{M_{1}}{M}$$

With:

- M_1_: mass in grams of 200 µL of tested EO.
- M: mass in grams of 200 ul of distilled water.

#### 3.4.2. Miscibility with Ethanol

This test was performed following the previous protocol of AFNOR standards [19]. After adding an equivalent volume of ethanol to the EO and stirring, the amount of ethanol needed to make the solution clear was noted.

#### 3.4.3. Freezing Point

The freezing point of the *M. rotundifolia* EO placed in the freezer is determined using a precision thermometer following the temperature decrease that occurs as the EO freezes, according to the AFNOR standards for EO analysis [19].

### 3.5. Chemical Profile Determination Using GC/MS-MS

To determine the chemical profile of volatile compounds in the EO extracted from *M. rotundifolia* leaves, a GC-MS/MS system consisting of a gas chromatograph coupled to a mass spectrometer (GC-MS/MS triple quadrupole TQ, SHIMADZU Corporation, GCMS TQ 8040NX, Vogelsang, Bellefonte, PA, USA) was operated. A capillary column (No-polar Rxi-5Sil MS—30 m × 0.25 mm ID × 0.25 µm, Restek, Bellefonte, PA, USA) was used. Helium was used as the carrier gas at a flow rate of 0.8 mL/min. The oven temperature was programmed from 50 to 200 °C, increasing at 5 °C/min. To identify the EO chemical components, the NIST library (version 2019, Shimadzu Corporation Vogelsang, Columbia, MD, USA) was used. The mass spectra obtained were evaluated using LabSolutions Insight™ data processing software, with a mass-to-charge range of 35 to 500 *m*/*z* for spectral scanning. The peak areas were calculated to determine the relative percentage of each identified chemical component of the analyzed EO.

### 3.6. Antibacterial Activity Assay

#### 3.6.1. Bacterial Strains

The selection of bacterial strains was based on a literature review of the most prevalent pathogenic strains associated with food poisoning [66]. The tested bacterial strains were obtained from Pasteur Institute (Casablanca, Morocco), and included four Gram+ and 2 Gram- bacteria, namely: *E. faecalis* CIP 103214, *S. aureus* ATCC 9144, *S.* Typhi CIP 5535, *B. cereus* ATCC 33019, *S. agalactiae* IPM 24842, and *P. alcalifaciens* CIP 82.90T.

#### 3.6.2. Inoculum Preparation

A bacterial suspension was prepared from pure colonies in 0.9% NaCl, and according to McFarland, the required optical density should be between 0.08 and 0.1 nm at a wavelength of 625 nm, representing 10^7^ to 10^8^ CFU/mL [67].

#### 3.6.3. Agar Disk Diffusion Assay

To test the antibacterial activity of EO against tested bacterial strains, a Mueller-Hinton agar medium was used following the method reported by Balouiri et al. [68]. Briefly, a sterile cotton swab was used to spread the previously prepared inoculum onto the medium. Afterwards, a sterile Whatman paper disc with a diameter of 6 mm was placed in the center of the plate. The disc was then covered with 10 µL of the tested EO and incubated at 37 °C for 24 h. The diameter of the inhibition zone was measured using a ruler. All antibacterial experiments were performed in triplicate (n = 3). Amoxicillin (25 µg/disc) and Penicillin (1 µL/disc) were selected as reference antibiotics (positive control) due to their well-established antibacterial activity and wide use as standards in the in vitro assays. They provide a reliable benchmark to compare the antibacterial effect of the EO against both Gram+ and Gram- bacterial strains [69,70], while the negative control discs contained only sterilized distilled water.

#### 3.6.4. Determination of Minimum Inhibitory Concentration (MIC)

The Minimum Inhibitory Concentration (MIC) was determined using the 96-well plate microdilution method following the instructions of Cosentino’s et al. [71], with minor modifications. Briefly, 50 µL of Nutrient Broth and 50 µL of EO at varying concentrations, diluted in 5% DMSO, were added to each well. Regarding the concentration gradient, a serial two-fold dilution was performed in the microplates. For this, each well initially contained 100 µL of nutrient broth. In the first well, 200 µL of the EO solution (prepared by diluting 200 µL of pure EO in 1 mL of 5% DMSO) were added, and serial dilutions were performed across the plate by transferring 100 µL from one well to the next. After mixing, 100 µL from each well were retained, forming the final series of concentrations prior to the addition of the bacterial inoculum. Subsequently, 25 µL of the bacterial suspension were added, and the plates were incubated for 18–20 h at 37 °C.

MIC determination was performed using a resazurin-based assay, allowing a clear visual discrimination between bacterial growth and inhibition. Wells in which the blue color of resazurin remained unchanged were considered as showing no bacterial growth, whereas a color change to pink indicated active bacterial metabolism. This visual criterion was used to define the MIC as the lowest concentration inhibiting bacterial growth.

For each bacterial strain, a positive control using streptomycin at a stock concentration of 1 mg/mL was included to confirm bacterial responsiveness, and a negative control without any treatment was performed under identical culture conditions to cheek the normal growth and viability of the bacterial strains. In addition, since the EO was dissolved in 5% DMSO for preparation, a solvent control using 5% DMSO alone was included to confirm that the solvent had no antibacterial activity. All these controls ensured that the observed inhibitory activity was solely attributable to the studied EO.

#### 3.6.5. Determination of Minimum Bactericidal Concentration (MBC)

Direct plating was performed from wells that exhibited growth inhibition onto MH agar medium using a sterile cotton swab after a 24 h incubation period at 37 °C to determine the MBC, which is the lowest EO concentration that completely inhibits bacterial growth. The antibacterial effect is thus classified as bacteriostatic or bactericidal depending on the MBC/MIC ratio. Bacteriostatic activity is defined as MBC/MIC > 4, while a bactericidal effect is defined as MBC/MIC ≤ 4 [72].

#### 3.6.6. Time-Kill Kinetics Assay

The time-kill kinetics of *M. rotundifolia* EO were evaluated against the tested bacterial strains following a standard viable count method reported by Di Rosario et al. [73]. Bacterial suspensions were prepared from fresh overnight cultures and adjusted to approximately 1 × 10^6^ CFU/mL in Mueller Hinton broth. The EO was added to obtain a final concentration corresponding to the previously determined MIC value. A negative growth control containing a bacterial suspension without the addition of EO was included in parallel. The inoculated tubes were incubated at 37 °C under aerobic conditions. At predetermined time intervals (0, 2, 4, and 8 h), aliquots of 100 µL were collected from each tube. Serial decimal dilutions were prepared in sterile physiological saline solution, and appropriate dilutions were spread onto Mueller Hinton agar plates. After incubation at 37 °C for 18–24 h, colonies were counted and the results were expressed as log_10_ CFU/mL.

### 3.7. Molecular Docking Simulation

#### 3.7.1. Molecular Geometry

DFT remains the most widely used method for the precise study of complex molecular systems. It enables efficient prediction of molecular properties from the geometry of a molecule [74]. Calculations and optimizations of products were performed using the B3LYP method combined with the 6-31G+(d,p) basis set, using the Gaussian 09 and Gauss View 5.0 software [75]. Global electronic properties, including the frontier molecular orbitals (LUMO and HOMO), the energy gap (ΔE), and various reactivity indices such as chemical potential (μ), chemical hardness (η), softness (S), global electrophilicity (ω), and global nucleophilicity (N), were determined. These indices, within the framework of conceptual density functional theory, are calculated using the following expressions: μ = ((EHOMO + ELUMO)/2), η = (EHOMO − ELUMO), S = 1/η, and ω = μ2/2η. Global nucleophilicity N is calculated relative to tetracyanoethylene N = E(HOMO(Nu)) − E(HOMO(TCE)) [76]. All optimized structures correspond to minima on the potential energy surface (characterized by positive vibrational frequencies). The electronic localization function (ELF) was used to analyze the distribution of electron density at the bonds and in the non-bonded regions [77]. Additionally, parameters such as the energy of frontier orbitals (HOMO and LUMO), as well as descriptors of chemical reactivity, are presented in Table 3.

#### 3.7.2. Molecular Docking’s in Silico Screening

Molecular docking with AutoDock Vina and the PyRx 0.8 tool [78] assessed the biological activity of the major components of the studied EO. Targeting the proteases of certain pathogenic bacteria including *B. cereus*, *E. faecalis*, *S.* Typhi, *S. aureus*, *S. agalactiae*, and *P. alcalifaciens* and, the study used AutoDockTools-1.5.6, for protein preparation by adding polar hydrogens, deleting water molecules, and applying Kollman charges [79]. By selecting poses with the lowest binding free energy from the nine produced, molecular docking enabled the prediction of the optimal binding configurations between the drugs and their targets [80]. Biovia Discovery Studio 2021 Client was then used to observe and illustrate the ligands’ intermolecular interactions with the active site amino acids [81]. Docking grids were centered on the co-crystallized ligand of each structure to target the active site:

- 3WT0 (*B. cereus*): 20 × 20 × 20 Å; center (4.53, −6.34, 60.01) Å.
- 5CDP (*S. aureus*): 20 × 20 × 20 Å; center (11.78, 50.11, 44.02) Å.
- 6J90 (*S.* Typhi): 24 × 24 × 24 Å; center (−12.65, 51.02, 28.42) Å.
- 4M7U (*E. faecalis*): 20.7911 × 15.7199 × 11.9981 Å; center (12.7148, −24.2497, 13.481) Å.
- 5Y2G (*S. agalactiae*): 20 × 20 × 20 Å; center (67.27, 165.24, −0.86) Å.
- 6LK2 (*P. alcalifaciens*):13.4173 × 17.7532 × 12.059 Å; center (5.05716, 5.77914, 56.1322) Å.
- 7P2N (DNA gyrase (GyrB)): 20 × 20 × 20 Å; center (−19.996750, −14.398875, 6.856083) Å.

#### 3.7.3. In Silico Assessment

In order to identify potential health risks at an early stage, the theoretical toxicological profile of the main chemical components of *M. rotundifolia* EO (Carvacrol, Piperitenone, Pulegone and Rotundifolone) was assessed using the pkCSM web platform, used exclusively as an in silico toxicological screening tool [69]. This platform allows several general toxicity indicators to be estimated, including mutagenicity, acute and chronic toxicity, and certain environmental toxicity parameters. In the context of food bioconservation, this approach aims to provide preliminary information on the potential compatibility and safety of the EO components.

### 3.8. Statistical Analysis

All experimental data were presented as mean ± standard deviation (SD) and analyzed using SPSS statistical software (version 26). A level of *p* < 0.05 was considered statistically significant. One-way ANOVA was used to evaluate differences between groups, followed by Tukey’s post hoc test for multiple comparisons. The Pearson correlation analysis was performed using the peakage::corrt and peakage::complot functions in the R environment to assess the relationship between chemical groups and strains.

## 4. Conclusions

The present investigation addressed the physicochemical properties, chemical profile, antibacterial activity, and the molecular interactions of *M. rotundifolia* EO components. A total of 27 compounds were identified and the main components included Rotundifolone, Carvacrol, Pulegone and Piperitenone. Experimental tests revealed that *M. rotundifolia* EO possessed significant antibacterial activity, which can be attributed to the oxygenated monoterpenes. This was further supported by molecular docking, which indicated that the most efficient inhibitor was against *B. cereus* (CMI and CMB values of 0.58 and 1.46 µL/mL, respectively). Toxicological analysis showed that the identified compounds are not carcinogenic and are not hepatotoxic. These results provide a scientific basis for future research exploring the potential applications of *M. rotundifolia* EO, particularly in food-related systems. However, further investigations, including stability studies, volatility assessment, interactions with food matrices, and in situ evaluations, are required to confirm its practical applicability.

## Figures and Tables

**Table 1:** Chemical composition of *M. rotundifolia EO*: major compounds and chemical classes identified by GC-MS/MS.

Major Compounds	Formula	RI	MW	RT	%
Rotundifolone	C_10_H_14_O_2_	1236	166	20.23	27.95
Carvacrol	C_10_H_14_O	1262	150	18.51	19.48
Piperitenone	C_10_H_14_O	1223	150	19.628	6.09
Cinerolon	C_10_H_14_O_2_	1426	166	21.176	4.73
Pulegone	C_10_H_16_O	1212	152	16.873	4.47
Cis-Piperitone epoxide	C_10_H_16_O_2_	1171	168	17.291	3.64
Caryophyllene	C_15_H_24_	1494	204	21.852	3.24
Oxygenated monoterpenes					72.45
Sesquiterpenes					16.7
Non-oxygenated monoterpenes					4.95

Note: RI: retention indice; MW: molecular weight; RT: retention time; %: relative percentage.

**Table 2:** Literature data from Maghreb countries (*Algeria*, *Morocco*, and *Tunisia*) reporting the antibacterial activity of *M. rotundifolia* EO against various Gram+ and Gram- bacterial strains.

Country	Bacterial Strains	Inhibition Diameter (mm)	Effect	Reference
*Algeria*				
	*Staphylococcus aureus*	26.26 ± 0.25	+++	[28]
	*Salmonella typhimurium*	14.16 ± 0.15	++	
	*Klebsiella pneumoniae*	14.33 ± 0.28	++	
	*Escherichia coli*	14.23 ± 0.18	++	
	*Listeria monocytogenes*	29.33 ± 0.57	+++	[29]
	*Staphylococcus aureus*	22.33 ± 2.08	+++	
	*Enterobacter aerogenes*	20 ± 1	+++	
	*Escherichia coli*	13 ± 1	+	
	*Pseudomonas aeruginosa*	10 ± 0.57	+	
	*Staphylococcus aureus*	17.33 ± 1.15	++	[30]
	*Bacillus subtilis*	30.00 ± 2.00	+++	
	*Salmonella enteritidis*	24.33 ± 2.08	+++	
	*Staphylococcus aureus CIP7625*	28.3 ± 0.28	+++	[27]
	*Streptococcus pyogenes CIPA22*	−	−	
	*Escherichia coli CIP54.8*	15.3 ± 0.20	++	
	*Pseudomonas aeruginosa CIPA22*	−	−	
	*Enterobacter* spp.	10	+	[31]
	*Escherichia coli*	24	+++	
	*Pseudomonas aeruginosa*	11	+	
	*Proteus mirabilis*	22	+++	
	Methicillin-resistant *Staphylococcus aureus ATCC 43300*	26.0 ± 1.0	+++	[32]
	*Staphylococcus aureus NCCB 9163*	20.0 ± 1.0	+++	
	*Bacillus subtilis ATCC6633*	19.6 ± 0.6	++	
	*Escherichia coli ATCC 25922*	11.0 ± 1.0	+	
	*Pseudomonas aeruginosa ATCC27853*	7.0 ± 0.0	−	
	*Klebsiella pneumoniae E47*	8.0 ± 0.0	−	
*Tunisia*				
	*Staphylococcus aureus*	24 ± 0.5	+++	[21]
	*Bacillus cereus*	23 ± 1.1	+++	
	*Escherichia coli*	34 ± 0.5	+++	
	*Salmonella typhimurium*	31 ± 1	+++	
*Morocco*				
	*Staphylococcus aureus*	10	+	[26,30]
	*Acinetobacter baumannii*	16	++	
	*Klebsiella pneumoniae*	10	+	
	*Salmonella sushi*	14	+	
	*Escherichia coli*	11	+	
	*Proteus mirabilis*	56	+++	
	*Pseudomonas aeruginosa*	−	−	

Note: (−) no activity; (+) low activity; (++) moderate activity; (+++) high activity.

**Table 3:** Values of total energies (in eV), HOMO-LUMO energies (in eV), chemical electronic potentials µ (in eV), electronegativity χ (in eV), global hardness η (in eV), dipole moments Md (in debye), electrophilicity indices ω (in eV), and nucleophilicity indices N (in eV) of the studied compounds.

Compound	Total Energy	E (HOMO)	E (LUMO)	µ	Χ	η	ω	N	S	Md
Carvacrol	−12,647.02	−6.077	−0.28	−3.18	3.18	5.79	0.87	3.33	0.172	1.48
Piperitenone	−12,646.21	−6.49	−1.65	−4.075	4.075	4.83	1.71	2.91	0.206	4.15
Pulegone	−12,679.28	−6.50	−1.40	−3.95	3.95	5.10	1.53	2.90	0.195	3.36
Rotundifolone	−14,692.48	−6.73	−1.68	−4.21	4.21	5.049	1.75	2.67	0.198	3.29

**Table 4:** The binding affinity of the main EO constituents toward the selected bacterial targets was estimated based on the Gibbs free energy (ΔG, kcal/mol) associated with the highest-scoring docking pose predicted by AutoDock Vina.

	Carvacrol	Piperitenone	Pulegone	Rotundifolone	Streptomycin
*B. cereus (3wt0)*	−6.3	−6.5	−6.1	**−6.8**	−7.2
*E. faecalis (4M7U)*	**−6.0**	−5.8	−5.9	**−6.0**	−6.9
*S.* Typhi *(6J90)*	−5.8	−6.0	**−6.5**	−6.2	−5.7
*S. aureus (5cdp)*	−6.1	−6.4	−6.2	**−6.8**	−8.9
*P. alcalifaciens (6lk2)*	−5.4	−5.6	**−6.0**	−5.9	−7.8
*S. agalactiae (5y2g)*	−6.7	**−7.2**	−7.0	**−7.2**	−8.9
*DNA gyrase: GyrB (7P2N)*	−6.1	**−6.2**	−6.0	−6.0	−6.5

**Table 5:** In silico toxicological profile of the main constituents of EO of *M. rotundifolia* relevant to food biopreservation.

Propriety	Carvacrol	Piperitenone	Pulegone	Rotundifolone
AMES toxicity	No	No	No	No
Oral rat acute Toxicity (LD50) (mol/kg)	1.828	1.65	1.838	1.913
Oral rat chronic Toxicity (LOAEL) (mg/kg b.w./day)	2.299	2.063	2.124	2.223
Hepatotoxicity	No	No	No	No
Skin Sensitization	Yes	Yes	Yes	Yes
*Tetrahymena pyriformis* toxicity (log μg/L)	0.699	0.394	0.355	−0.264
Minnow toxicity (log mM)	0.83	1.241	1.432	1.727

## Data Availability

The raw data supporting the conclusions of this article will be made available by the authors on request.

## Supplementary Materials

The following supporting information can be downloaded at: https://www.mdpi.com/article/10.3390/ijms27083527/s1.
