# Supplementary material for: Integrated Evaluation of Mentha rotundifolia (L.) Huds Essential Oil: Physicochemical Characterization, Antibacterial Effect and In Silico ADMET Prediction
Source: Int J Mol Sci. 2026 Apr 15;27(8):3527. doi: 10.3390/ijms27083527 (PMC13115922; doi:10.3390/ijms27083527)
Supplement: Supplementary file 1 [file ijms-27-03527-s001.zip › ijms-4202845-supplementary.pdf]

Supplementary files (Table S1 and Figure S1)

Table S1. Statistical parameters of the correlation analysis between major chemical compounds and MIC/MBC values.

Tested strains		Rotundifolone	Carvacrol	Piperitenone	Cinerolon	Pulegone	Cis-Piperitone epoxide	Caryophyllene
B. cereus	Pearson correlation	-1.000**	0.655	0.500	-1.000**	-0.500	-0.982	-0.500
	Sig. (Two-tailed)	0.000	0.546	0.667	0.000	0.667	0.121	0.667
	N (Sample size)	3	3	3	3	3	3	3
E. faecalis	Pearson correlation	-0.500	0.982	-0.500	-0.500	0.500	-0.655	-1.000**
	Sig. (Two-tailed)	0.667	0.121	0.667	0.667	0.667	0.546	0.000
	N (Sample size)	3	3	3	3	3	3	3
P. alcalifaciens	Pearson correlation	-0.500	0.982	-0.500	-0.500	0.500	-0.655	-1.000**
	Sig. (Two-tailed)	0.667	0.121	0.667	0.667	0.667	0.546	0.000
	N (Sample size)	3	3	3	3	3	3	3
S. agalactiae	Pearson correlation	-0.500	0.982	-0.500	-0.500	0.500	-0.655	-1.000**

	Sig. (Two-tailed)	0.667	0.121	0.667	0.667	0.667	0.546	0.000
	N (Sample size)	3	3	3	3	3	3	3
S. aureus	Pearson correlation	-0.500	0.982	-0.500	-0.500	0.500	-0.655	-1.000**
	Sig. (Two-tailed)	0.667	0.121	0.667	0.667	0.667	0.546	0.000
	N (Sample size)	3	3	3	3	3	3	3
S. typhi	Pearson correlation	-0.500	0.982	-0.500	-0.500	0.500	-0.655	-1.000**
	Sig. (Two-tailed)	0.667	0.121	0.667	0.667	0.667	0.546	0.000
	N (Sample size)	3	3	3	3	3	3	3

Note*: Pearson correlation coefficients were calculated between the major chemical compounds and MIC/MBC values. Sig. (two-tailed) indicates the bilateral significance level, and N represents the number of observations used for each correlation.

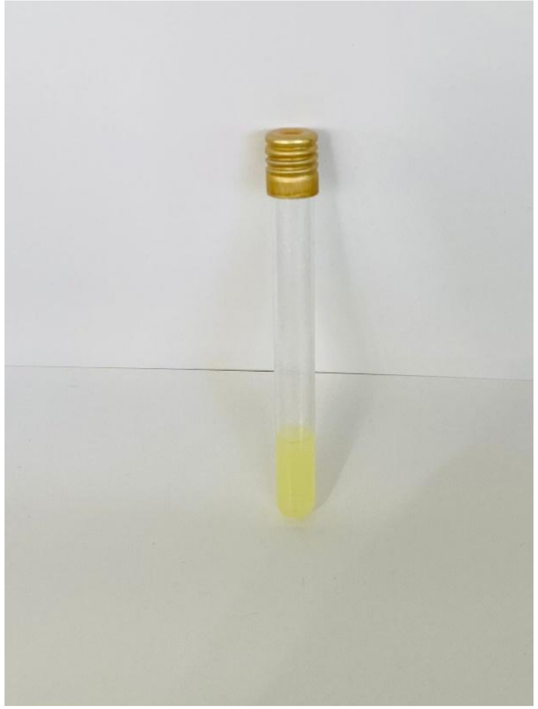

Figure S1. Physical appearance of the extracted essential oil.
